# The Effect of Vitamin C on Parathyroid Hormone in Patients on Hemodialysis With Secondary Hyperparathyroidism: A Double Blind, Placebo-Controlled Study

**DOI:** 10.5812/numonthly.12404

**Published:** 2013-11-13

**Authors:** Vajihe Biniaz, Eghlim Nemati, Ali Tayebi, Mehdi Sadeghi Shermeh, Abbas Ebadi

**Affiliations:** 1 Department of Medical Surgical Nursing, Baqiyatallah University of Medical Sciences, Tehran, IR Iran; 2 Nephrology and Urology Department, Baqiyatallah University of Medical Sciences, Tehran, IR Iran

**Keywords:** Renal Dialysis, Parathyroid Hormone, Hyperparathyroidism, Secondary, Ascorbic Acid

## Abstract

**Background:**

Secondary hyperparathyroidism (SHPT) is a prevalent disorder in patients with chronic kidney disease. It is proffered that there is a contradictory relation between serum level of vitamin C and parathyroid hormone (PTH) in hemodialysis patients with secondary hyperparathyroidism.

**Objectives:**

The goal of this study was to assess the effects of the supplemental vitamin C on parathyroid hormone among hemodialysis patients with secondary hyperparathyroidism.

**Patients and Methods:**

This randomized, placebo-controlled, double-blind and parallel-group trial was conducted on 82 hemodialysis patients with serum levels of PTH more than 200 pg/mL. In intervention group, 250 mg vitamin C was injected three times a week for 8 weeks in a row immediately at the end of each dialysis session via the intravenous route. In the control group, same term of placebo saline was injected.

**Results:**

The mean of serum PTH was 699.81 (± 318.8) and 596.03 (± 410.7) pg/mL in intervention and control groups respectively at baseline (reference range, 6 to 66 pg/mL), and at the end of study it changed to 441.4 and 424.6 in these groups. The values of serum Calcium and Phosphate did not significantly change during the study (8.4 ± 0.6 mg/dL versus 8.1 ± 0.8 mg/dL, P = 0.39; 5.89 ± 1.7 mg/dL versus 5.9 ± 1.9 mg/dL, P = 0.08, respectively).

**Conclusions:**

This study finding does not warranted therapeutic effect of vitamin C on secondary hyperparathyroidism.

## 1. Background

Secondary hyperparathyroidism (SHPT) is one of the most prevalent disorders in patients with chronic kidney disease (CKD) (1), which most hemodialysis patients are faced with (up to 50%) (2). Although SHPT is a result of increased parathyroid hormone (PTH) synthesis due to phosphorus accumulation and hypocalcemia (3), other factors such as impairment of vitamin D metabolism and reduced PTH renal clearance (4) may also exacerbate it (5). Hypocalcemia (Low serum levels of ionized calcium) frequently happens as a result of calcium inadequate response to parathyroid hormone (1).

High levels of PTH, considered as a uremic toxin (6), can stimulate accelerated bone absorption and reabsorption, and cause bone demineralization and renal osteodystrophy (7). The bones which become demineralizated are structurally fragile, easily broken, and not resistant to any shock. In this stage, there is higher risk of fractures (8). This mineral metabolism abnormality is also one of the main risk factors for ectopic calcification and cardiovascular events in hemodialysis patients (3).

To prevent the main problems created by SHPT such as cardiovascular mortality and fractures (9), it is necessary to examine and analyze different methods to reduce parathyroid hormone in patients on hemodialysis (10). Whereas there is a potential link between the occurrence of SHPT and low vitamin C levels in conformity with some studies (3), vitamin C supplementation is probably a way to reduce parathyroid hormone with fewer side effects (1).

In low serum levels of vitamin C, calcium-sensing receptors may become resistant to PTH influence (11). Vitamin C increases the response to PTH at these receptors by increasing the cyclic adenosine monophosphate and reducing PTH through it (12). 

Hemodialysis patients are at risk for low levels of serum vitamin C (13). Vitamin C is a water-soluble vitamin which can be reduced by regular hemodialysis (14). Dietary restrictions, following fear related to hyperkalemia (15), concern oxalosis, wasting several hundred mg of vitamin C during one session of hemodialysis (16), and fast catabolism lead to less usage than actual necessity of vitamin C in hemodialysis patients. Although normal range of serum vitamin C is 30-60 μM, most hemodialysis patients have serum levels of vitamin C less than 10 μM , and even some of them have less than 2 μM (17). Moreover, chronic inflammation due to the release of inflammatory mediators in hemodialysis patients leads to less production of essential antioxidants and more oxidative stress (18), and it causes an increase in free radicals associated with vitamin C deficiency as an important antioxidant (19).

Even if the intake of 60 to 100 mg of vitamin C for health maintenance in an individual with normal kidney function is sufficient, it may not be adequate in a hemodialysis patient due to mentioned reasons. The recommended amounts for the intake of vitamin C in hemodialysis patients are 100-200 mg per day which almost none of patients on hemodialysis use it (3).

Hitherto tested different methods for the treatment of SHPT in hemodialysis patients. Accomplished studies were often with a small sample size, without placebo, randomization and control group. Despite excessive number of patients receiving dialysis and transplant in Iran (exceeded 50,000 in 2011) (20), no examination has been sufficiently reported to show the influences of vitamin C on SHPT.

## 2. Objectives

This study was performed to evaluate the vitamin C effects on parathyroid hormone among hemodialysis patients with secondary hyperparathyroidism.

## 3. Patients and Methods

This randomized, placebo-controlled, double-blind and parallel-group trial was conducted from October 2012 to January 2013 on 82 ESRD patients undergoing maintenance dialysis in two hemodialysis units in Baqiyatallah and Chamran hospitals of Tehran, Iran. The inclusion criteria were: age ≥ 18 years, regular recourse for maintenance hemodialysis three sessions per week, receiving dialysis for ≥ 3 months, serum PTH level > 200 pg/mL, and not taking vitamin C at least from 3 months ago.

Of 82 randomized patients, 6 were excluded from the study due to transmission to other dialysis centers, being infected by active infections, getting cancers, death, or their own refusal, and only 76 patients completed the study (37 person in case, and 39 person in control group).

Both groups were the same in clinical particulars including age, sex, weight, marital and employment status, length and session of hemodialysis, and smoking. The length of hemodialysis in all patients was approximately 4 hours and its occasions were 3 times a week with resemble kt/V. Although high-flux dialysis membranes are more efficient in the removal of biointact PTH than low-flux membranes (1), some of the patients were dialyzed by low-flux membranes due to physiologic problems such as hypotension and cramp during dialysis. Blood flow and dialysis solution flow rate were 250 mL/min to 300 mL/min and 500 mL/min, respectively. The KT/V had no change during our study. Dialysis solution calcium (2.5 mmol/L) did not change till the end of the study.

Prior ethical approval was obtained from the institutional ethical committee at Baqiyatallah University of Medical Sciences, Tehran, Iran. A justification letter was sent to two hemodialysis units to gain permission to collect the data granted by these units. Verbal and written consents were obtained from all those who participated in the study. Written consents were obtained after informing each participant about the study purposes, the “confidentiality” of their information, and the possibility to refuse the test procedure at any stage of it. 

Required laboratory parameters including serum biointact PTH, P, and Ca levels were measured at the beginning and the end of the intervention. The PTH blood sample was taken via the venous line at the beginning of hemodialysis session. Serum PTH, Ca, and P levels were respectively determined by enzyme-linked immunosorbent, endpoint, and fixed-time assay.

In intervention group, 250 mg Vitamin C was injected three times a week for 8 weeks in a row immediately at the end of each hemodialysis session via the intravenous route. In the control group, same term of placebo saline was injected. Although the recommended amount for vitamin C intake in patients on hemodialysis is 100-200 mg per day (1), only 250 mg vitamin C three times a week – which was lower than the safe dosage recommended by NIH (21) – was prescribed to prevent oxalosis.

The data was analyzed by SPSS software (version 18). The χ2-test, T-test and ANOVA were used in this study. The researcher decided to receive only a 5% error in rejecting the full hypothesis at the 95% confidence interval. The significance level was put at 0.05.

## 4. Results

Demographic data of the participants showed that, among 76 respondents, 46 (60.5%) patients were male and 30 (39.5%) female. The mean age of participants was 60.6 years (SD 11.47) (maximum and minimum range was 29-81 years).

Tables 1 and 2 exhibited the demographic Characteristics of the Study Population. The mean level of serum PTH decreased to 441.42 (± 311.6), and 424.6 (± 386.4) after the intervention in study and control groups respectively. 

**Table 1.** Baseline Qualitative Characteristics of the Respondents

Variables	Group, No. (%)		χ2-test
	Intervention	Control	
**Gender**			0.18
Male	20 (26.3)	26 (34.2)	
Female	17 (22.4)	13 (17.1)	
**Marriage**			0.29
Married	31 (40.8)	33 (43.4)	
Single	2 (2.6)	0	
Widow	6 (7.9)	4 (5.3)	
**Education**			0.81
Primary-secondary	31 (40.9)	29 (38.1)	
College/university-level	8 (10.5)	8 (10.5)	
**Employment**		0	0.43
Jobless	17 (22.4)	12 (15.8)	
Employed	3 (3.9)	4 (5.3)	
Retired	17 (22.4)	23 (30.2)	
**Smoking**			0.48
Yes	1 (1.3)	0	
No	36 (47.4)	39 (51.3)	
**Nephropathy cause**			0.45
HTN^a^	14 (18.4)	17 (22.4)	
DM^a^	4 (5.3)	6 (7.9)	
HTN and DM	9 (11.8)	9 (11.8)	
Others	10 (13.1)	7 (9.2)	

^a^Abbreviation: DM, diabetes mellitus; HTN, hypertention.

**Table 2.** Baseline Quantitative Characteristics of the Respondents

Variable	Group, Mean (SD)		T-test
	Intervention	Control	
**Age, y**	60.32 (12.2)	60.97 (10.9)	0.8
**Dialysis vintage, mo**	63.27 (67.8)	40.4 (32.8)	0.63
**Body weight, kg**	68.08 (9.4)	72.1 (9.7)	0.07
**Serum parameters**			
Intact parathyroid hormone, pg/mL	699.8 (318.8)	596 (410.3)	0.22
Calcium, mg/dL	8.43(0.49)	8.37 (0.71)	0.35
Phosphate, mg/dL	6.008 (1.76)	5.787 (1.66)	0.57

There was no significant correlation between the serum levels of PTH and vitamin C prescription. PTH level alterations in different stages of study are summarized in Table 3. Serum Calcium and Phosphate levels did not significantly change during the study (8.4 ± 0.6 mg/dL versus 8.1 ± 0.8 mg/dL, P = 0.39; 5.89 ± 1.7 mg/dL versus 5.9 ± 1.9 mg/dL, P = 0.08, respectively). 

**Table 3.** Biointact Parathyroid Hormone (PTH) Levels

Groups	PTH, pg/mL	
	Baseline	2 Months
**Intervention**	699.8 (318.85)	441.42 (311.61)
**Control**	596.03 (410.3)	424.65 (386.42)
**P-Value**	0.22	0.83

## 5. Discussion

The results of the present study demonstrated that vitamin C supplementation cannot decrease serum level of PTH significantly.

According to data, the incidence of CKD is higher in men and people older than 45 years. In this study, the mean age of patients was also 60.66 (SD 11.47) years, and most of them (60.5%) were male and 90.8% of patients were older than 45 years. These results are supported by earlier studies.

In other studies more than 50% hemodialysis patients were jobless, but in our study only 3.9% of the patients were unemployed, and the rest (96.1%) were retired or employed. This could indicate that the government and insurance companies provide appropriate support for hemodialysis patients in Iran. Like similar studies, in this study diabetes and hypertension were the most common causes of nephropathy (diabetes and hypertension 77.7%). 

In 2008, Richter proposed that there is an inverse correlation between serum level of vitamin C and biointact PTH (3). He measured serum vitamin C level, while prescribing no vitamin C analogues. 

Similarly, in 2011 Sanadgol reported that vitamin C is able to reduce biointact PTH level. Although there were no placebo and control groups in his study, he confirmed that there is an inverse correlation between vitamin C and SHPT in hemodialysis patients (1).

In 2011, Sanadgol and his colleagues measured the mean level of biointact PTH after a prescription of 200 mg of vitamin C, three times a week for 3 months, and explained that the mean of serum PTH was notably reduced at the end of the first month after the prescription of vitamin C. But this influence became gradually weaker after 2 months, so that serum level of PTH increased in 3 months; however, it was still lower than the baseline level. They stated that the reason of this finding may be associated with decreased calcium-sensing sensitivity of receptors to vitamin C.

In spite of Sanadgol study, we observed no significant association between serum levels of PTH and vitamin C. It can be associated with sample size diversity (21 versus 76), usage of placebo, randomization, and control group in our study.

At the initiation of the plan, we selected patients with serum PTH levels more than 200 pg/mL, and randomly divided them into two parallel groups. We prescribed 250 mg intravenous vitamin C immediately after hemodialysis for 2 months, and then assessed the PTH level changes. None of our sampled patients recently used vitamin C supplements. Nevertheless, in our study the level of serum PTH was not measured at the end of the first month. The mean of serum PTH decreased at the end of the second month in the intervention group (699.8 versus 441.4). It can be demonstrated that vitamin C influences on the serum level of PTH. But there was a decrease in serum level of PTH in the control group too; however, it was not comparable with the reduction in the intervention group (441.4 versus 424.6). The main cause of the observed diminution in serum levels of PTH at control group is unknown, but it may be associated with what is called “placebo effect”. Also, we should have examined the serum levels of PTH at the termination of the first month after prescription.

Not measuring plasma level of vitamin C before, and after the study, and not specifying the patients who had vitamin C deficiency before study were the limitations in our study which prevented the capability to generalize the findings. Removing these limitations was not a feasible option due to the financial costs, and the limitations of the laboratories capable of providing the circumstance for this test.

Conclusions: This study finding does not warranted therapeutic effect of vitamin C on secondary hyperparathyroidism. Although serum level of PTH decreased in intervention group with supplemental vitamin C, this decrease was observed in placebo group too. Therefore in this study we did not observe any significant association between vitamin C supplementation and secondary hyperparathyroidism. 

## References

[1] Sanadgol H, Bayani M, Mohammadi M, Bayani B, Mashhadi MA (2011). Effect of vitamin C on parathyroid hormone in hemodialysis patients with mild to moderate secondary hyperparathyroidism.. Iran J Kidney Dis..

[2] Gorriz JL, Molina P, Bover J, Barril G, Martin-de Francisco AL, Caravaca F (2013). Characteristics of bone mineral metabolism in patients with stage 3-5 chronic kidney disease not on dialysis: results of the OSERCE study.. Nefrologia..

[3] Richter A, Kuhlmann MK, Seibert E, Kotanko P, Levin NW, Handelman GJ (2008). Vitamin C deficiency and secondary hyperparathyroidism in chronic haemodialysis patients.. Nephrol Dial Transplant..

[4] Brossard JH, Lepage R, Cardinal H, Roy L, Rousseau L, Dorais C (2000). Influence of glomerular filtration rate on non-(1-84) parathyroid hormone (PTH) detected by intact PTH assays.. Clin Chem..

[5] Massry SG (1977). Is parathyroid hormone a uremic toxin?. Nephron..

[6] Polzin DJ, Osborne CA, Jacob F, Ross S, Ettinger SJ, Feldman EC (2000). Chronic renal failure.. Textbook of veterinary internal medicine..

[7] Weiner DE, Tighiouart H, Vlagopoulos PT, Griffith JL, Salem DN, Levey AS (2005). Effects of anemia and left ventricular hypertrophy on cardiovascular disease in patients with chronic kidney disease.. J Am Soc Nephrol..

[8] Martin KJ, Gonzalez EA (2001). Strategies to minimize bone disease in renal failure.. Am J Kidney Dis..

[9] Ganesh SK, Stack AG, Levin NW, Hulbert-Shearon T, Port FK (2001). Association of elevated serum PO(4), Ca x PO(4) product, and parathyroid hormone with cardiac mortality risk in chronic hemodialysis patients.. J Am Soc Nephrol..

[10] Mahdavi-Mazdeh M, Zamyadi M, Norouzi S, Heidary Rouchi A (2007). Management of calcium and phosphorus metabolism in hemodialysis patients in Tehran Province, Iran.. Iran J Kidney Dis..

[11] McCauley LK, Koh AJ, Beecher CA, Cui Y, Rosol TJ, Franceschi RT (1996). PTH/PTHrP receptor is temporally regulated during osteoblast differentiation and is associated with collagen synthesis.. J Cell Biochem..

[12] Pearce SH, Thakker RV (1997). The calcium-sensing receptor: insights into extracellular calcium homeostasis in health and disease.. J Endocrinol..

[13] Deicher R, Ziai F, Bieglmayer C, Schillinger M, Horl WH (2005). Low total vitamin C plasma level is a risk factor for cardiovascular morbidity and mortality in hemodialysis patients.. J Am Soc Nephrol..

[14] Bohm V, Tiroke K, Schneider S, Sperschneider H, Stein G, Bitsch R (1997). Vitamin C status of patients with chronic renal failure, dialysis patients and patients after renal transplantation.. Int J Vitam Nutr Res..

[15] Durose CL, Holdsworth M, Watson V, Przygrodzka F (2004). Knowledge of dietary restrictions and the medical consequences of noncompliance by patients on hemodialysis are not predictive of dietary compliance.. J Am Diet Assoc..

[16] Morena M, Cristol JP, Bosc JY, Tetta C, Forret G, Leger CL (2002). Convective and diffusive losses of vitamin C during haemodiafiltration session: a contributive factor to oxidative stress in haemodialysis patients.. Nephrol Dial Transplant..

[17] Handelman GJ (2007). Vitamin C deficiency in dialysis patients--are we perceiving the tip of an iceberg?. Nephrol Dial Transplant..

[18] Farsi Z (2007). [Rabeteye esteres oksidativ va bimarihaye koleye]. Faslnameye amouzeshie daneshkade parastari-e Bagheyatallah.. Educ Quar J Nurs Facult Baghyatalah Univ Med Sci..

[19] Weissinger EM, Nguyen-Khoa T, Fumeron C, Saltiel C, Walden M, Kaiser T (2006). Effects of oral vitamin C supplementation in hemodialysis patients: a proteomic assessment.. Proteomics..

[20] Aghazamani M (2011.). Renal patients increase 20% yearly.. http://www.salamatiran.com/NSite/FullStory.

[21] Dietary Supplement Fact Sheet: Vitamin C..

